# Characterization of Soil Suppressiveness to Root-Knot Nematodes in Organic Horticulture in Plastic Greenhouse

**DOI:** 10.3389/fpls.2016.00164

**Published:** 2016-02-17

**Authors:** Ariadna Giné, Marc Carrasquilla, Maira Martínez-Alonso, Núria Gaju, Francisco J. Sorribas

**Affiliations:** ^1^Departament d'Enginyeria Agroalimentària i Biotecnologia, Universitat Politècnica de CatalunyaCastelldefels, Spain; ^2^Departament de Genètica i Microbiologia, Facultat de Biociències, Universitat Autònoma de BarcelonaBellaterra, Spain

**Keywords:** antagonistic potential of soil, biological control, biodiversity, DGGE fingerprints, *Meloidogyne* spp., *Pochonia chlamydosporia*, vegetable crops

## Abstract

The fluctuation of *Meloidogyne* population density and the percentage of fungal egg parasitism were determined from July 2011 to July 2013 in two commercial organic vegetable production sites (M10.23 and M10.55) in plastic greenhouses, located in northeastern Spain, in order to know the level of soil suppressiveness. Fungal parasites were identified by molecular methods. In parallel, pot tests characterized the level of soil suppressiveness and the fungal species growing from the eggs. In addition, the egg parasitic ability of 10 fungal isolates per site was also assessed. The genetic profiles of fungal and bacterial populations from M10.23 and M10.55 soils were obtained by Denaturing Gradient Gel Electrophoresis (DGGE), and compared with a non-suppressive soil (M10.33). In M10.23, *Meloidogyne* population in soil decreased progressively throughout the rotation zucchini, tomato, and radish or spinach. The percentage of egg parasitism was 54.7% in zucchini crop, the only one in which eggs were detected. *Pochonia chlamydosporia* was the only fungal species isolated. In M10.55, nematode densities peaked at the end of the spring-summer crops (tomato, zucchini, and cucumber), but disease severity was lower than expected (0.2–6.3). The percentage of fungal egg parasitism ranged from 3 to 84.5% in these crops. The results in pot tests confirmed the suppressiveness of the M10.23 and M10.55 soils against *Meloidogyne*. The number of eggs per plant and the reproduction factor of the population were reduced (*P* < 0.05) in both non-sterilized soils compared to the sterilized ones after one nematode generation. *P. chlamydosporia* was the only fungus isolated from *Meloidogyne* eggs. In *in vitro* tests, *P. chlamydosporia* isolates were able to parasitize *Meloidogyne* eggs from 50 to 97% irrespective of the site. DGGE fingerprints revealed a high diversity in the microbial populations analyzed. Furthermore, both bacterial and fungal genetic patterns differentiated suppressive from non-suppressive soils, but the former showed a higher degree of similarity between both suppressive soils than the later.

## Introduction

Root-knot nematode (RKN), *Meloidogyne* spp., is the most harmful plant-parasitic nematode on vegetable crops in the world (Sasser and Freckman, 1987). In Spain, RKN are present in all horticulture production areas (Melgarejo et al., 2010) causing economical losses. Estimation of the maximum yield losses on important vegetable crops include: 88% for cucumber, 60% for tomato, 39% for zucchini, 37% for watermelon, and 30% for lettuce (Verdejo-Lucas et al., 1994; Sorribas et al., 2005; Talavera et al., 2009; Giné et al., 2014; López-Gómez et al., 2014; Vela et al., 2014).

Soil fumigants and nematicides are the most popular control methods (Talavera et al., 2012). However, the Directive 2009/128/EC from the European Commission promotes the use of non-chemical methods based on integrated pest management strategies in order to reduce the use of pesticides.

A sustainable production system uses environmentally friendly alternatives to preserve and enhance beneficial organisms, which represents the antagonistic potential. Soils with high antagonistic potential lead to suppression of soil borne pathogens. In a suppressive soil, pathogens do not establish, persist, or establish but cause little or no damage (Baker and Cook, 1974). Suppressive soils have already been described for many soil pathogens (Weller et al., 2002) including plant parasitic nematodes (Timper, 2011). Suppressive soils to cyst nematodes and RKN have been intensively studied. In such soils, fungal parasites were responsible of suppression in cereal (Kerry, 1980), sugar beet (Westphal and Becker, 1999, 2001), and soybean cyst nematodes (Chen, 2007), as well as RKN (Pyrowolakis et al., 2002; Adam et al., 2014). However, the suppression mechanisms are not well understood. Janvier et al. (2007) summarized the biotic and abiotic factors related to soil suppressiveness. Among them, the soil microbiota plays an important role (Weller et al., 2002), being essential to sustain biological productivity (Garbeva et al., 2004). Soil microbial diversity changes depending on the type of plant, soil, and management, and the interaction of microorganisms with those other factors can lead to the soil's disease suppressiveness (Garbeva et al., 2004). The study of microbial communities can be done by culture-independent methods, such as denaturing gradient gel electrophoresis (DGGE) (Muyzer et al., 2004), which allows the analysis of the total microbial structure of the soil, including the microorganisms that cannot be recovered by cultivation (Smalla and Heuer, 2006).

In Spain there are no reports of soils suppressive to RKN, despite the occurrence of antagonists of the nematode (Verdejo-Lucas et al., 1997, 2002, 2013; Olivares-Bernabeu and López-Llorca, 2002). In 2010, 10 commercial organic production sites were sampled in the northeastern horticultural growing area of Spain to assess the occurrence of fungal egg parasites of RKN and the percentage of parasitized eggs. Fungal egg parasites occurred in all sampled sites, and mean percent of parasitized eggs was 36.2 (Giné et al., 2012). In some of those sites, growers did not use any specific control measures against *Meloidogyne*, although attenuated disease symptoms were observed, mainly at the end of the spring-summer crops. Then, could be considered that some of those soils could be suppressive to RKN despite be intensively perturbed agrosystems, this is, with several crops per season and favorable climatic conditions that enable nematodes' development. Furthermore, as far as we know, there is little knowledge about the fluctuation of soil suppressiveness during the cropping sequences in commercial farms or the microbial profiles of RKN suppressive soils. Thus, two sites were selected in order to (i) determine the fluctuation of the RKN population's densities and the percentage of fungal egg parasitism along 2 years, (ii) assess soil suppressiveness in pot test, (iii) know the parasitic ability to RKN eggs of the fungal isolates from each soil, and (iv) compare microbial profiles between these two soils and a conducive one.

## Materials and methods

### Sites

Two commercial organic horticultural production sites, M10.23 and M10.55, cropped in plastic greenhouses were selected from a previous study (Giné et al., 2012) considering that the percentage of fungal egg parasitism was similar to the average obtained from all organic production sites sampled (36.2%). Both sites are located at the Tarragona province (northeastern Spain). Physicochemical properties and enzymatic activity of soils are presented in Table 1, and the rotation sequences conducted for both sites appear in Table 2. Soil at M10.23 was infested with *Meloidogyne javanica*; fertilization was done using a mixture of composted sheep and chicken manure at a rate of 2 kg m^−2^ that was incorporated into the soil just before transplanting each crop. Weed management was done by flaming and mechanically. Soil at M10.55 was infested with *M. javanica* and *Meloidogyne incognita* at a rate 10:1; fertilization was done with composted sheep manure at a rate of 1.7 kg m^−2^ that was also incorporated just before transplanting each crop. Mustard was grown as a cover crop planted in summer, just at the end of the spring crop, and incorporated as green manure 2 weeks before transplanting the autumn crop. Weeds were managed mechanically.

**Table 1:** **Physicochemical properties and enzymatic activity of soil of two vegetable production sites managed organically (M10.23 and M10.55) and an integrated production site (M10.33) in plastic greenhouses at the beginning of the study**.

**Variable**	**Sites**		
	**M10.23**	**M10.55**	**M10.33**
Sand (%)	45	68	50
Silt (%)	40	0	20
Clay (%)	15	32	30
Soil texture (USDA)	Loam	Sandy clay loam	Sandy clay loam
pH	8.3	8.1	7.9
Organic matter (w/w)	5.8	2.5	1.6
Electric conductivity (μS/cm)	276	1069	2030
B (ppm)	2.8	1.1	4.6
Exchangeable Ca (meq 100 g^−1^)	17.3	18.2	8.8
Available Ca (meq 100 g^−1^)	17.2	19.0	14.6
Lime	3.8	4.1	4.4
Cation exchange capacity (meq 100 g^−1^)	41.2	25.7	13.7
Cu (ppm)	3.6	2.5	3.5
Available P (ppm)	379.4	75.8	107.6
Fe (ppm)	11.4	5.0	5.0
Exchangeable Mg (meq 100 g^−1^)	4.0	3.0	2.3
Available Mg (meq 100 g^−1^)	5.0	3.7	4.7
Mn (ppm)	64.0	2.5	148.0
N (ppm)	2329	1498	865
Exchangeable K (meq 100 g^−1^)	1.2	0.7	0.5
Available K (meq 100 g^−1^)	1.9	0.7	1.0
C/N	14.4	9.7	10.5
Exchangeable Na (meq 100 g^−1^)	0.3	0.5	0.3
Available Na (meq 100 g^−1^)	1.0	3.2	3.0
Zn (ppm)	20.6	2.5	81.0
Ca + Mg/K	18.0	31.5	21.4
P/N	0.2	0.1	0.1
Fluorescein diacetate hydrolysis (μg fluorescein h^−1^ × g soil)	5.5	1.0	2.0
b-glucosaminidase (μmols *p*-nitrophenol h^−1^ × g soil)	0.4	0.1	0.1
Urease (μmols N–NH_4_ h^−1^ × g soil)	1.6	0.9	0.1
Protease (μg tyrosine h^−1^ × g soil)	4.5	12.4	8.7

**Table 2:** ***Meloidogyne* population densities in soil at planting (*Pi*) and at the end of the crop (*Pf*), galling index, number of eggs on roots, and percentage of egg parasitism in two commercial organic vegetable productions sites in plastic greenhouse during two consecutive years (2011–2013)**.

**Site**	**Crop^a^**	**Date**	**N° of juveniles 250 cm^−3^ soil**		**Galling index**	**N° of eggs g root^−1^**	**Parasitized eggs (%)**
			***Pi***	***Pf***			
M10.23	Zucchini cv. Dundoo	07/2011–11/2011	2951±487	61±15	1.6±0.3	1301±530	54.7±13.9
	Tomato cv. Royesta (R)	01/2012–07/2012	61±15	0±0	0±0	0±0	Nem
	Radish cv. Saxa	11/2012–02/2013	9±9	3±3	0±0	0±0	Nem
	Spinach cv. Gigante de invierno	11/2012–02/2013	4±4	9±3	0±0	0±0	Nem
	Fallow	02/2013–07/2013	6±3	15±12	Na	Na	Na
M10.55	Tomato cv. Lladó (R)	02/2011–07/2011	238±62	1013±883	0.2±0.1	41±26	3.0±0.04
	Zucchini cv. Dundoo	07/2011–11/2011	1013±883	1351±238	3.0±0.4	1870±478	84.5±3.6
	Lettuce cv. Maravilla	11/2011–03/2012	1351±238	81±17	0.1±0.1	0±0	Nem
	Cucumber cv. Dasher II	03/2012–06/2012	81±17	1329±505	6.3±1.0	6026±1165	71.7±2.7
	Mustard cv. Caliente 109	06/2012–09/2012	1329±505	40±18	Na	Na	Na
	Lettuce cv. Maravilla	09/2012–11/2012	40±18	56±5	2.2±0.2	999±645	0.2±0.2
	Tomato cv. Caramba (R)	02/2013–07/2013	19±6	126±30	1.3±0.8	206±115	16.0±10.6

*Data are mean ± standard error of four replications. Galling index on a scale from 0 to 10, where 0 = complete and healthy root system and 10 = plants and roots dead (Zeck, 1971)*.

a *R, Resistant cultivar; Na, Not available; Nem, Not egg masses*.

The commercial production site M10.33 was selected as non-suppressive soil due to its history on *Meloidogyne* infestation and disease severity on cucumber, pea, and tomato. At the end of those crops, fungal egg parasites were recovered at low percentage, 4.1% after cucumber crop (Giné et al., 2012), and 0% after the pea and tomato crops (data not shown). The grower managed RKN by biosolarization after the spring-summer crop. The site was conducted under integrated production in plastic greenhouse located in the province of Barcelona (northeastern Spain). Physicochemical properties and enzymatic activity of soils are also presented in Table 1. Fertilization was based on pellets of composted manure combined with chemical fertilizers. Weeds were managed mechanically. The soil of this site was used in the DGGE analysis for comparison between microbial communities of soils.

### Fluctuation of RKN population densities

Composite soil samples were collected at the beginning and at the end of each crop to determine initial (*Pi*) and final (*Pf*) nematode population densities. Each plastic greenhouse was divided in four plots of 75 and 82 m^2^ at the M10.23 and the M10.55 sites, respectively. Individual samples consisting of 20 soil cores were taken from the first 30 cm of soil with a soil auger (2.5 cm diameter) from each plot. Soil cores were mixed thoroughly and sieved through a 4-mm aperture screen to remove stones and separate roots from soil. RKN juveniles (J2) were extracted from two 250-cm^3^ soil subsamples using the sieving and centrifugation-flotation method (Jenkins, 1964). J2 were counted and expressed as J2 per 250 cm^3^ of soil. The reproduction rate of RKN in each crop was calculated as *Pf* /*Pi* ratio. At the end of each crop, eight plants per plot were randomly collected and removed from the ground with a pitchfork; damage caused by RKN in the root system was rated for galling based on a scale from 0 to 10, where 0 = complete and healthy root system and 10 = plants and roots dead (Zeck, 1971). Roots were carefully washed free of soil, mixed, chopped, and root-knot nematode eggs extracted from two10 g-subsamples by macerating them for 10 min in a blender containing a 1% NaOCl solution (Hussey and Barker, 1973). Eggs were counted and expressed per g of root.

Soil temperature and soil water content from each site were recorded at 60 min intervals with temperature probes (5TM, Decagon devices, Inc., Pullman, WA, USA) placed at 15 cm depth.

### Fungal egg parasitism

At the end of each crop, fungal egg parasites of RKN were isolated according to the de Leij and Kerry (1991) procedure modified by Verdejo-Lucas et al. (2002). Briefly, per each plot, 10–20 egg masses were handpicked from roots and placed in a watchglass containing sterile distilled water. The outer part of the gelatinous matrix was removed from the egg masses with tweezers to eliminate potential surface colonizers. Egg masses were then placed in an Eppendorf microcentrifuge tube containing 1 ml of sterile distilled water. Eggs were dispersed from the egg masses using a pestle and 333 μl-aliquots of the eggs' suspension were spread onto each of three replicated Petri dishes (9-cm diameter) containing a growth restricting medium (streptomycin, 50 mg l^−1^; chloramphenicol, 50 mg l^−1^; chlortetracycline, 50 mg l^−1^; Rose Bengal, 50 mg l^−1^; triton, 1 ml l^−1^; and 1% agar) (Lopez-Llorca and Duncan, 1986). Plates were incubated at 25 ± 0.5°C. Number of parasitized eggs was recorded after 24 and 48 h under a dissecting microscope and percentage of parasitism was then calculated as the number of parasitized eggs per plate/number of eggs per plate. Eggs were considered parasitized if fungal hyphae grew from inside. At least, 20 parasitized eggs per plot and crop were individually transferred to corn meal agar (CMA) to establish pure cultures of the fungi. Fungal isolates were stored in 1% (w/v) water-agar slants, as well as lyophilized and stored at 4°C.

### Fungal parasites characterization

Identification of fungal species isolated at the end of the first crop was carried out by PCR amplification and sequencing of the internal transcribed spacers (ITSs) of the rDNA regions. DNA was extracted from 50 mg of mycelium collected from single spore cultures on potato dextrose agar (PDA) using the E.Z.N.A kit® Plant MiniPrep (Omega Bio-Tek) according to the protocol described by the manufacturer. The PCR reaction was performed in 25 μl mix that contained 1 μl of the DNA extraction, 10.5 μl MiliQ water (Qiagen), 12.5 μl Taq PCR Master Mix (Qiagen) and 0.5 μl of each primer (5 pmol), ITS1F (Gardes and Bruns, 1993) and ITS4 (White et al., 1990). PCR conditions were the same as those described in the original studies (White et al., 1990). PCR products were cleaned using MinElute PCR Purification Kit (Qiagen) and sequenced by Secugen (Madrid, Spain). DNA sequences were analyzed using the BLAST database (July 2013) and assigned to the reference isolate sequences with the highest bit score. Identification of fungal isolates from eggs produced on the rest of crops was carried out according morphological characters (Gams, 1988).

### Soil suppressiveness against RKN in pot tests

Two experiments were carried out in 2012. Experiment 1 was conducted from March 27 to June 10 [907 degree-day (DD), 10°C basal temperature and thermal constant between 600 and 700 DD over the basal temperature; (Ferris et al., 1985)] with soil samples taken in January 2012. Experiment 2 was carried out from August 9 to October 23 (1092 DD, 10°C basal temperature and thermal constant between 600 and 700 DD over the basal temperature) with soil samples taken in July 2012. Both experiments were conducted using the same procedure. A soil sample was taken from the first 30 cm of soil with a hoe. Sample consisted of 48 soil cores (12 per plot). Soil was mixed thoroughly and passed through a 5-mesh sieve to remove stones and separate roots from soil. A part of soil was sterilized at 121°C during 1 h and the procedure was repeated after 1 day. The rest of soil was stored at 4°C until the experiment was carried out. Sterilized soil was mixed with steam-sterilized sand at a ratio 1:1 (dry w: dry w) to avoid soil compaction and improve plant growth. The same procedure was carried out with non-sterilized soil. After that, RKN juveniles were extracted from two 500-cm^3^ subsamples of both sterilized and non-sterilized soil mixtures using Baermann trays (Whitehead and Hemming, 1965) maintained at 27 ± 2°C for a week to determine the level of nematode inoculum at the beginning of the experiments. Thereafter, soil was placed in 3-L pots and a susceptible tomato cv. Durinta was transplanted into each pot at three true developed leaves stage. Nematode inoculum consisted of juveniles emerged from eggs that were extracted from tomato roots by the Hussey and Barker (1973) procedure and placed in Baermann trays (Whitehead and Hemming, 1965) for a week at 27 ± 2°C. Soil was inoculated with *M. incognita* J2 to achieve a total of 3000 J2 per plant, which was added in two opposite holes, 3 cm deep, made in the soil at 2 cm from the stem of the plants.

Ten replicate pots were prepared per each soil mixture, site, and experiment. Plants were arranged at random on a greenhouse bench, were irrigated as needed and fertilized with a slow-release fertilizer (15N + 10P + 12K + 2MgO + microelements). Soil temperatures and soil water content at 8 cm depth was recorded at 30 min interval during the experiments.

At the end of the experiments, plants were removed from pots. Roots were washed with tap water to remove soil particles and gently dry before determine fresh weight. Galling index was estimated according to Zeck scale (1971). To determine percentage of egg parasitism, three egg masses were handpicked from individual plants growth in both sterilized and non-sterilized soils and processed according to the method described previously. Fungi growing from eggs were isolated and identified as previously described. Eggs were extracted from roots by Hussey and Barker (1973) method, and reproduction factor was calculated considering *Pi* as number of juveniles inoculated, and *Pf* number of non-parasitized eggs per plant (Sorribas et al., 2003).

### Parasitism of fungal isolates against RKN eggs

Five single-spore culture isolates of *Pochonia chlamydosporia* coming from each pot test and site were assessed for fungal egg parasitism. Single 5 mm-diameter plugs from the margin of the colony growing on PDA were transferred to the center of plates containing 1% water agar (WA) and incubated at 25°C in the dark for 2 weeks. Sterilized RKN eggs used as inoculum were obtained according to the procedure of Verdejo et al. (1988) modified. Briefly, 30 *M. incognita* eggs masses coming from tomato roots were handpicked and placed in a sterile conical centrifuge tube containing 1 ml of 4% NaOCl solution. The egg suspension was shaken during 4 min at 30 s intervals, and finally diluted 10 times with sterile distilled water. Egg suspension was left undisturbed for 30 min to allow deposition. After that, sterilized nematode eggs were spread axenically around 1-cm apart from the margin of the colony using a Pasteur pipette. Plates were incubated at 25°C in the dark for 1 week. Eggs surrounded by a dense fungal colony were considered as parasitized and validated by observation under the light microscope (Lopez-Llorca et al., 2002). Percentage of egg parasitism was calculated as described previously. Three replicate plates were prepared per each fungal isolate and experiment.

### DGGE analysis of fungal and bacterial soil community DNA

Fungal and bacterial profiles from M10.23 and M10.55 soils were obtained by DGGE, and compared to a commercial vegetable production site, M10.33, managed under integrated production, but with low percentage of fungal egg parasitism (4.1%) (Giné et al., 2012). Soil samples used for this study were taken in February 2013.

DNA extraction of soil samples was carried out using the Ultraclean Soil DNA Kit (MoBio Laboratories, Carlsbad, CA, USA) according to the manufacturer's protocol, using 0.25 g of soil. DNA extractions were performed from each composite soil sample. The quantity and quality of the extracted DNA was checked by agarose gel electrophoresis and by spectrophotometer measurement at wavelength 260 and 280 nm. All DNA samples were stored at −20°C for further analyses.

Fungal DNA was amplified using a nested approach, where primers EF4/ITS4 (Gardes and Bruns, 1993) amplifies 18S rDNA and ribosomal ITS regions in a first PCR, and this product is then used as template in a second PCR applying primers ITS1f-GC/ITS2 (White et al., 1990). Bacterial DNA was amplified using the universal bacterial primers 341F-GC and 907R (Muyzer et al., 2004). The PCR mixture and conditions were the same as those described in the original studies. PCR products were analyzed for size and quantity by agarose gel electrophoresis and stained with ethidium bromide. DGGE analyses were carried out using a D-Code Universal Detection System (Bio-Rad Laboratories, Richmond, CA, USA).

Nine hundred nanograms of PCR product were loaded onto 8% (w/v) polyacrylamide gels (40% acrylamide/bis solution, 37.5:1, Bio-Rad) with denaturing gradients ranging from 10 to 50% for the fungal DNA and 20 to 70% for the bacterial DNA (100% denaturants defined like 7 M urea and 40% v/v deionized formamide) (Schäfer and Muyzer, 2001). Electrophoresis was performed in 1 × Tris-acetate-EDTA (TAE) buffer, at 60°C. The gel with fungal DNA was run for 16 h at 75 V, while the gel with bacterial DNA was run for 16 h at 80 V. Gels were stained with ethidium bromide (0.5 μg/ml), and inspected under UV illumination and photographed. Prominent bands were excised from the gels, reamplified, and then purified using the PCR Clean up Kit (MoBio Laboratories) for subsequent sequencing.

Sequencing reactions were performed by Macrogen (South Korea) using the Big Dye Terminator v3.1 sequencing kit; reactions were run in an automatic capillary type ABI 3730XL analyzer-96. Sequences were first screened to detect potential chimeric artifacts using the Chimera.uchime program in Mothur 1.33.3 (http://www.mothur.org/wiki/Download_mothur) (Edgar et al., 2011) and then compared to those deposited in the GenBank nucleotide database using the BLAST program (Tatusova and Madden, 1999; Maidak et al., 2001).

### Statistical analyses

Statistical analyses were carried out with the SAS system software V9.2 (SAS Institute Inc., Cary, NC, USA). Variables were transformed when required to log_10_ (x + 1) or arcsine square root (x + 0.5). Data from pot experiments to assess soil suppressiveness were compared between experiments and site by *t*-Student test, using the *t*-test procedure, and were pooled together as replications of a single experiment because no differences (*P* > 0.05) were found. Then, data were submitted to *t*-Student test to compare between sterilized and non-sterilized mixture soil per each site. Data from experiments conducted to determine the ability of fungal isolates to parasitize RKN eggs were submitted to analysis of variance using the general linear model (proc glm) to compare the parasitic capability between isolates per site. When the analyses were significant (*P* ≤ 0.05), the means were separated according to the least significant difference (LSD) test.

DGGE images were analyzed using the InfoQuest™FP 4.5 software (Bio-Rad Laboratories, Richmond, CA, USA). Similarities of the DGGE profiles were calculated based on the Dice coefficient and dendrograms were obtained using the UPGMA clustering algorithm. A band position tolerance of 0.5% was used. Band patterns were normalized using the marker lanes as reference, allowing the comparison among samples loaded on different DGGE gels. The number of DGGE bands in each fingerprint was used as a measure of the apparent fungal and bacterial richness (S). Shannon Index was used as a measure of genetic diversity, and was calculated as H = pi ln pi, where pi is the relative intensity of each DGGE band. Evenness (E) was calculated as E = ln (S). Diversity variables were submitted to nonparametric analysis of variance (proc npar1way) using Wilcoxon rank sum test.

## Results

### Fluctuation of RKN population densities and percentage of fungal egg parasitism

**Figure 1:**
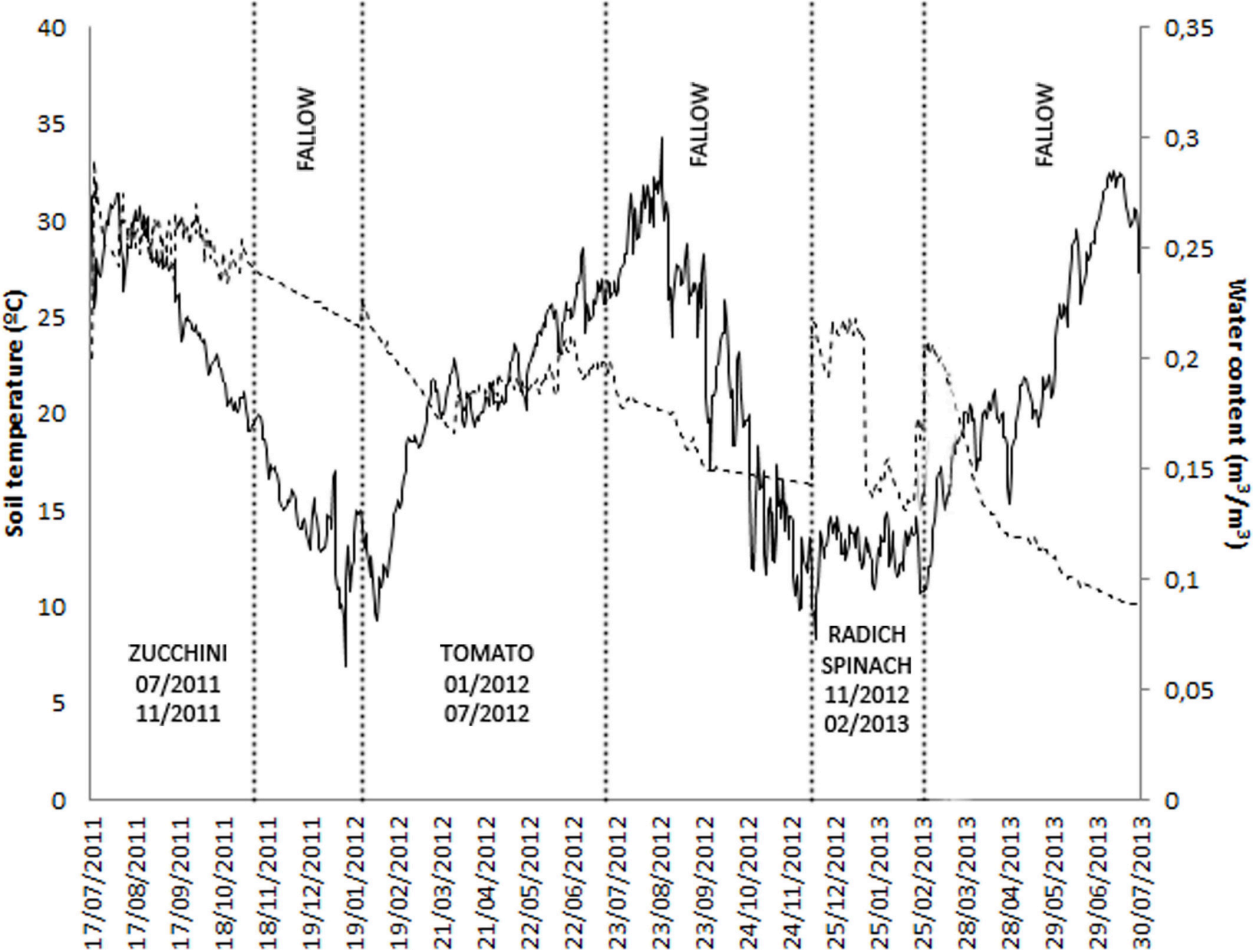
**Fluctuation of mean daily soil temperature, soil water content, and crop rotation sequence in site M10.23**.

**Figure 2:**
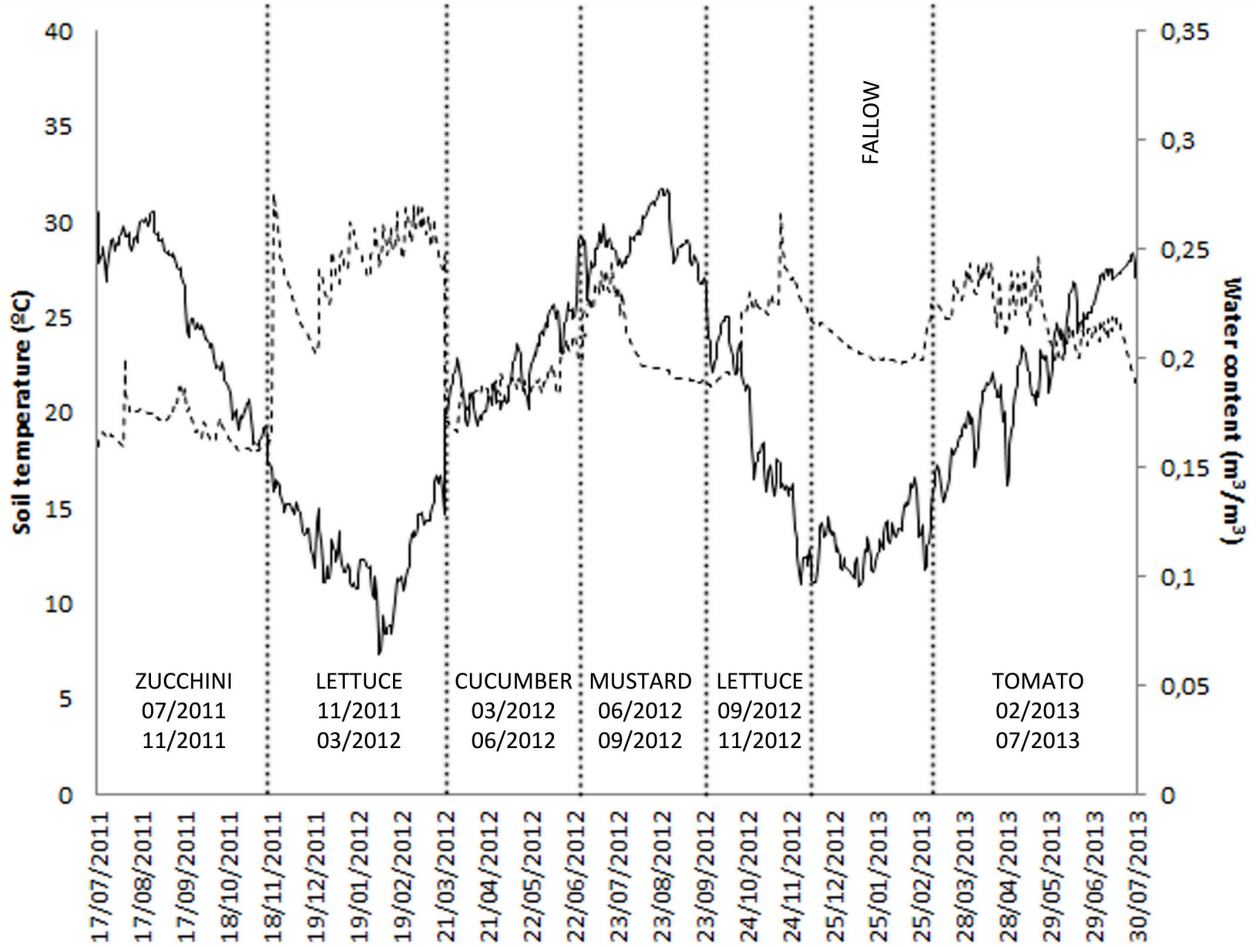
**Fluctuation of mean daily soil temperature, soil water content, and crop rotation sequence in site M10.55**.

Daily soil temperature and water content of soil, as well as crop rotation sequences in sites M10.23 and M10.55, are presented in Figures 1, 2, respectively.

In M10.23, nematode population in soil decreased progressively from 2951 J2 250 cm^−3^ of soil in July 2011 to 15 J2 250 cm^−3^ of soil in July 2013, all throughout the rotation zucchini-resistant tomato-fallow-radish/spinach-fallow. At planting the winter crops (radish and spinach), nematode densities were below 10 J2 250 cm^−3^ of soil, and did not increase at the end of the crop. For these two crops, no galls were observed in the roots and no eggs were recovered (Table 2). Maximum densities of eggs per g root were recovered from the RKN susceptible zucchini cv. Dundoo cropped during summer, but not from the resistant tomato cv. Royesta. Disease severity in zucchini ranged from 0 to 4, while no galls were observed on the tomato roots. Fungal egg parasitism was only detected in zucchini (54.7%) in which root infection occurred and egg masses were produced (Table 2). *P. chlamydosporia* was the only fungal species isolated.

In M10.55, densities of nematodes in soil peaked at the end of spring-summer crops. Population densities at planting of the susceptible cucumber cv. Dasher II and the zucchini cv. Dundoo crops were 81 and 1013 J2 250 cm^−3^ of soil, respectively, but disease severity was less than expected [galling index (GI) of 6.3 and 3.0, respectively (Table 2)]. High percentage of fungal egg parasitism was recorded after cultivation of the susceptible cucurbit crops (84.5 and 71.7%), it was low after the cropping of resistant tomatoes (16%), and not apparent in the winter lettuce crop (0%), in which no egg masses were produced (Table 2). Nematode densities decreased after incorporation of the mustard cover crop into the soil, but survivors were able to infect roots (GI = 2.0) of the following crop of lettuce cultivated from September to November 2013, and to produce eggs, some of which were parasitized (0.2%). Again, *P. chlamydosporia* was the only fungal egg parasite recovered.

### Soil suppressiveness against RKN in pot test

Minimum and maximum soil temperatures ranged from 18.6 to 25.7°C (21.9 ± 1.8°C, mean ± standard deviation) in experiment 1, and from 17.9 to 30.7°C (24.7°C ± 3.4°C) in experiment 2. Water content of soil ranged from 0.14 to 0.31 w^3^/w^3^ (0.22 ± 0.04 w^3^/w^3^) and from 0.15 to 0.26 w^3^/w^3^ (0.21 ± 0.03 w^3^/w^3^) in experiment 1 and 2, respectively.

Fungal egg parasites were recovered only from non-sterilized soils, being *P. chlamydosporia* the only fungal species identified. Eggs were parasitized at a rate of 24.8% in non-sterilized soil from site M10.23, and 70.9% from site M10.55 (Table 3). In non-sterilized M10.23 soil, fewer (*P* < 0.05) eggs per plant (73.30%), lower reproduction factor (73.91%), and less disease severity (17.07%) were recorded compared to the sterilized soil according to the Abbott's formula. In addition, less (*P* < 0.05) tomato fresh root weight (61.96%) was also recorded. Similar results were obtained with the non-sterilized M10.55 soil, in which the number of eggs per plant, reproduction factor and tomato fresh root weight were 61.43, 66.67, and 45.07% less (*P* < 0.05) than in the sterilized one, although disease severity did not differ (*P* > 0.05) (Table 3).

**Table 3:** **Effect of soil sterilization or not- sterilization of sites M10.23 and M10.55 on *Meloidogyne* densities on roots, reproduction factor, galling index, percentage of egg parasitism and fresh root weight of tomato cv. Durinta inoculated with 3000 juveniles per pot after completion of one nematode generation**.

**Site**	**Soil mixture^a^**	**Fresh root weight (g)**	**N° of eggs (× 10^3^)/plant^b^**	**Reproduction factor^c^**	**Galling index^d^**	**Parasitized eggs (%)**
M10.23	Sterilized	16.3±1.3^*^	41.2±13.4^*^	13.8±4.5^*^	4.1±0.2^*^	0
	Non-sterilized	6.2±0.5	11.0±3.0	3.6±1.0	3.4±0.2	24.8±1.7
M10.55	Sterilized	14.2±1.5^*^	40.7±8.8^*^	12.6±2.9^*^	4.3±0.2	0
	Non-sterilized	7.8±0.6	15.7±2.8	4.2±0.7	3.8±0.1	70.9±2.0

Data are mean ± standard error of 20 replications. Data within the same column and site followed by

* *indicates a significant difference between soil treatment at P < 0.05 according to the Student's t -test*.

a *Sterilized soil mixture, 50% sterilized soil + 50% sterilized sand; Non-sterilized soil mixture, 50% non-sterilized soil + 50% sterilized sand*.

b *Parasitized eggs excluded*.

c *Number of non-parasitized eggs per plant/initial population density*.

d *Galling index on a scale from 0 to 10, where 0 = complete and healthy root system and 10 = plants and roots dead (Zeck, 1971)*.

### Parasitism of fungal isolates against RKN eggs

*P. chlamydosporia* isolates from site M10.23 parasitized between 55.5 and 97.4% of the RKN eggs, and those from site M10.55 between 56.5 and 93.7%. In both sites, 3 out 10 fungal isolates parasitized more than 90% of the RKN eggs (Table 4).

**Table 4:** **Percentage of parasitized eggs of *Meloidogyne* spp. by isolates of *P. chlamydosporia* in *in vitro* test**.

**Site**	**Assay. Isolate^a^**	**Parasitized eggs (%)**
M10.23	C1.1	55.5±1.0 d
	C1.2	66.5±18.7 bcd
	C1.3	83.7±7.3 abcd
	C1.4	60.6±10.7 cd
	C1.5	86.5±3.9 abc
	C2.1	82.9±2.7 abcd
	C2.2	97.4±1.8 a
	C2.3	88.0±4.8 abc
	C2.4	90.7±5.6 ab
	C2.5	93.9±2.1 ab
M10.55	H1.1	56.5±6.5 f
	H1.2	64.0±5.6 ef
	H1.3	65.1±5.9 ef
	H1.4	76.7±1.7 de
	H1.5	82.6±4.0 cd
	H2.1	93.7±1.7 ab
	H2.2	89.0±0.7 abc
	H2.3	91.1±0.3 abc
	H2.2	93.3±4.3 abc
	H2.5	86.3±2.4 bcd

*Data are mean ± standard error of three replications. Data within the same column and site followed by the same letter did not differ (P < 0.05) according to the LSD test*.

a *Single-spore isolates of P. chlamydosporia isolated at the end of both pot assay, in June 10 (1), and October 23 (2) 2012*.

### DGGE analysis of fungal and bacterial communities

**Figure 3:**
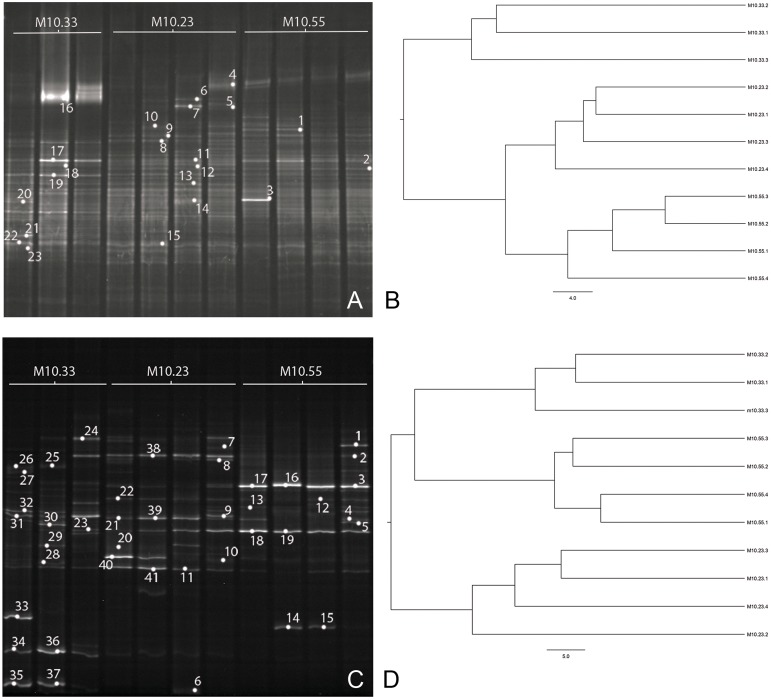
**DGGE profiles of 16S rRNA fragments of bacteria (A) and partial fungal ITS sequences (C) from DNA sample replicates of one non-suppressive (M10.33) and two suppressive (M10.23 and M10.55) soils**. Bands marked with numbers correspond to the bands excised from the gel and sequenced. Cluster dendrograms based on UPGMA algorithm show similarity among DGGE band patterns of bacteria **(B)** and fungi **(D)**. Bar indicates percentage of divergence.

Band profiles obtained by the DGGE of bacterial and fungal rDNA amplified fragments and the DGGE fingerprints cluster analysis are shown in Figure 3. The 16S rRNA-DGGE analysis (Figure 3A) revealed composite banding patterns reflecting a high microbial diversity. Conversely, the ITS rDNA-DGGE analysis (Figure 3C) showed a lower diversity in the fungal communities. Two first-order clusters were clearly differentiated by the UPGMA analysis of the DGGE fingerprints, both in the bacterial and the fungal communities of the soils. These first-order clusters were identified at a similarity score of 53 and 65% for the fungal and the bacterial communities, respectively. Regarding the bacterial community, the first-order cluster differentiated non-suppressive M10.33 soil from M10.23 and M10.55 suppressive soils, and the second-order subcluster (75.5% similarity) differentiated between both suppressive soils (Figure 3B). Concerning the fungal communities, M10.23 soil was clearly differentiated from the rest in the first-order clusters, and M10.55 and M10.33 soils were grouped in a second-order subcluster (56.5% similarity) (Figure 3D). The bacterial and fungal genetic diversity was evaluated based on the number of DGGE bands and their relative intensity. Diversity variables for the bacterial communities did not differ between soils (Shannon–Wiener *P* = 0.12; richness *P* = 0.73; evenness *P* = 0.09), but some of them did for the fungal communities. The Shannon–Wiener index and the evenness in soil M10.55 differed (*P* = 0.05 and *P* = 0.03, respectively) from M10.33 but not from M10.23 soils. However, richness was similar between soils (*P* = 0.45).

In Figures 3A,C, the bands marked with numbers correspond to the dominant bands that were extracted from the DGGE gels and sequenced. Tables 5, 6 show the sequenced bands, their similarity values compared to the closest related GenBank sequences, and their phylogenetic affiliations. Sequence similarity values compared to previously reported sequences were more than 93.5% in all cases. The majority of the 23 bacterial sequences belonged to the phylum Bacteroidetes (65.2%) followed by Proteobacteria (17.4%) (Table 5). Regarding fungi, the 41 sequences fell into three taxonomic groups. On average, Ascomycota (56.1%) was the most abundant phylogenetic groups, followed by Basidiomycota (31.7%) (Table 6).

**Table 5:** **DGGE bands sequenced, band length, associated GenBank accession numbers, and phylogenetic affiliation from profiles of bacterial populations**.

**Phylotype**			**Phylogenetic affiliation**		
**Band code^a^**	**Sequence length (bp)**	**Accession code**	**Taxonomic linage^b^(Phylum, Class, Order, Family, Genus)**	**Closest match^c^ (accession no.)**	**Similarity (%)^d^**
B1	336	KT991569	Bacteroidetes (100), Sphingobcteria (83), Sphingobacteriales (83)	Uncultured bacterium clone (KM155241)	94.4
B2	368	KT991570	Bacteroidetes (100), Sphingobcteria (100), Sphingobacteriales (100)	Uncultured *Sphingobacterium* sp clone (KM155241)	98.9
B3	536	KT991571	Cyanobacteria/Chloroplast (100)	Uncultured Streptophyta clone (JF703638)	99.8
B4	551	KT991572	Bacteroidetes (100), Flavobacteria (100), Flavobacteriales (100), Flavobacteriaceae (100), *Flavobacterium* (100)	*Flavobacterium* sp. (JN650574)	100
B5	519	KT991573	Bacteroidetes (100), Flavobacteria (100), Flavobacteriales (100), Flavobacteriaceae (100), *Chryseobacterium* (100)	*Chryseobacterium* sp. (KJ482798)	100
B6	570	KT991574	Bacteroidetes (100), Sphingobcteria (100), Sphingobacteriales (100), KD3-93 (100)	Uncultured Bacteroidetes (AM116744)	98.5
B7	556	KT991575	Bacteroidetes (100), Sphingobcteria (100), Sphingobacteriales (100), KD3-93 (100)	Uncultured bacterium clone (JF176318)	97.9
B8	547	KT991576	Bacteroidetes (100), Sphingobcteria (100), Sphingobacteriales (100), Cytophagaceae (100), *Flexibacter* (100)	Uncultured bacterium clone (KJ909017)	99.6
B9	541	KT991577	Bacteroidetes (100), Sphingobcteria (100), Sphingobacteriales (100), Cytophagaceae (100), *Flexibacter* (100)	Uncultured Flexibacteriaceae bacterium (FM209167)	99.4
B10	442	KT991578	Bacteroidetes, Sphingobcteria, Sphingobacteriales, Cytophagaceae (100), *Flexibacter* (100)	Uncultured Bacteroidetes bacterium (KJ024617)	98.0
B11	510	KT991579	Bacteroidetes (100), Sphingobcteria (100), Sphingobacteriales (100), Cytophagaceae (96), *Flexibacter* (89)	Uncultured Bacteroidetes bacterium (HF564268)	99.0
B12	511	KT991580	Bacteroidetes (100), Sphingobcteria (100), Sphingobacteriales (100), Cytophagaceae (100), *Flexibacter* (94)	Uncultured Bacteroidetes bacterium (HF564268)	99.8
B13	449	KT991581	Proteobacteria (100), Gammaproteobacteria (100), Xanthomonadales (98). Sinobacteriaceae (98), *Steroidobacter* (96)	Uncultured bacterium clone (GQ263704)	95.8
B14	519	KT991582	Proteobacteria (100), Gammaproteobacteria (100), Xanthomonadales (100), Xanthomonadaceae (100), *Lysobacter* (99)	*Lysobacter* sp. MHS036 (DQ993327)	97.5
B15	485	KT991583	Proteobacteria (100), Alphaproteobacteria (100), Rhizobiales (100), Methylobacteriaceae (100), *Methylobacterium* (100)	*Methylobacterium radiotolerans* (LC026013)	100
B16	490	KT991584	Bacteriodetes (100), Flavobacteria (100), Flavobacteriales (100); Flavobacteriaceae (100)	*Winogradskyella rapida* (KF009869)	93.5
B17	518	KT991585	Bacteroidetes (100), Sphingobcteria (100), Sphingobacteriales (100), Cytophagaceae (100), *Flexibacter* (100)	Uncultured Sphingobacteriales bacterium (KF733506)	99.6
B18	484	KT991586	Bacteroidetes (100), Sphingobcteria (100), Sphingobacteriales (100), Cytophagaceae (99), *Flexibacter* (99)	Uncultured Bacteroidetes bacterium (HF564295)	98.6
B19	513	KT991587	Bacteroidetes (100), Sphingobcteria (97), Sphingobacteriales (97), Cytophagaceae (94)	Uncultured Sphingobacteriales bacterium (AM936482)	99.8
B20	514	KT991588	Firmicutes (100), Bacilli (100), Bacillales (96), Bacillaceae (82)	*Marinococcus halophilus* (HF678777)	99.8
B21	472	KT991589	Unclassified Chloroflexi	Uncultured bacterium clone (HQ697759)	100
B22	494	KT991590	Proteobacteria (100), Alphaproteobacteria (100), Rhizobiales (100), Methylobacteriaceae (100), *Methylobacterium* (100)	*Methylobacterium mesophilicum* (KP293855)	100
B23	487	KT991591	Acidobacteria (100), Acidobacteria (100), Acidobacteriales (100), Acidobacteriaceae (100), Candidatus *Solibacter* (100)	Uncultured bacterium clone (JQ654947)	99.6

a *Band numbers correspond to those presented in Figure 3A for bacterial samples*.

b *Taxonomic string with bootstrap values (in parentheses), generated in mothur using SILVA database reference file release 119*.

c *Closest relative according to INSA (International Nucleotide Sequence Database)*.

d *Percentage sequence similarity with closest INSA using BLAST tool*.

**Table 6:** **DGGE bands sequenced, band length, associated GenBank accession numbers, and phylogenetic affiliation from profiles of fungal populations**.

**Phylotype**			**Phylogenetic affiliation**		
**Band code^a^**	**Sequence length (bp)**	**Accession code**	**Taxonomic linage^b^(Phylum, Class, Order, Family, Genus)**	**Closest match^c^(accession no.)**	**Similarity (%)^d^**
F1	231	KT991592	Ascomycota (100), Pezizomycetes (100), Pezizales (100), Pyronemataceae (100), *Pseudaleuria* (100)	Uncultured fungus clone (JF432996)	100
F2	219	KT991593	Ascomycota (100), Pezizomycetes (98), unclassified_Pezizomycetes_order (69)	Uncultured fungus clone (JX323746)	97.72
F3	173	KT991594	Ascomycota (100), Pezizomycetes (99), unclassified_Pezizomycetes_order (75)	Uncultured fungus clone (JX323746)	97.70
F4	189	KT991595	Ascomycota (100), Sordariomycetes (100), Hypocreales (99), Hypocreales_family_incertae_sedis (60), *Fusarium* (60)	*Fusarium equiseti* (KM246255)	98.4
F5	165	KT991596	Ascomycota (100), Dothideomycetes (89), Pleosporales (89), Sporormiaceae (84), *Preussia* (74)	Uncultured fungus clone (JX340328)	100
F6	251	KT991597	Ascomycota (100), Eurotiomycetes (100), Onigenales (100), Arthrodermataceae (100), *Ctenomyces* (100)	Uncultured fungus clone (JX349691)	99.2
F7	186	KT991598	Fungi_phylum_incertae_sedis (100), Fungi_class_incertae_sedis (100), Mortierellales (87), Mortierellaceae (85), *Mortierella* (74)	Uncultured fungus clone (JX377362)	97.85
F8	192	KT991599	Fungi_phylum_incertae_sedis (100), Fungi_class_incertae_sedis (100), Mortierellales (99), Mortierellaceae (98), *Mortierella* (98)	Uncultured fungus clone (JX345268)	99.48
F9	169	KT991600	Ascomycota (100), Dothideomycetes (100), Capnodiales (100), Capnodiales_family_incertae_sedis (100), *Cladosporium* (100)	*Graphiopsis chlorocephala* (JN116693)	100
F10	180	KT991601	Fungi_phylum_incertae_sedis (100), Fungi_class_incertae_sedis (100), Mortierellales (99), Mortierellaceae (94), *Mortierella* (94)	Uncultured fungus clone (JX387233)	98.9
F11	210	KT991602	Ascomycota (100), Sordariomycetes (100), Hypocreales (100), Hypocreales_family_incertae_sedis (100), *Stachybotrys* (100)	Uncultured *Stachybotrys* clone (KF493978)	100
F12	201	KT991603	Ascomycota (99), Sordariomycetes (96), Microascales (73), Microascaceae (73), *Pseudallescheria* (69)	Uncultured *Pseudallescheria* clone (KM108739)	99.5
F13	173	KT991604	Ascomycota (100), Sordariomycetes (98), Microascales (74), Microascaceae (74), *Pseudallescheria* (66)	Uncultured *Pseudallescheria* clone (KM108739)	100
F14	290	KT991605	Basidiomycota (100), Agaricomycetes (100), Agaricales (100), Psathyrellaceae (94), *Psathyrella* (60)	Uncultured fungus clone (GQ225128)	100
F15	292	KT991606	Basidiomycota (100), Agaricomycetes (100), Agaricales (100), Psathyrellaceae (100), *Psathyrella* (64)	Uncultured fungus clone (GQ225128)	100
F16	249	KT991607	Ascomycota (87), Pezizomycetes (78), Pezizales (76), Pyronemataceae (74), *Heydenia* (50)	Pezizaceae sp (JQ775581)	98.35
F17	220	KT991608	Ascomycota (100), Pezizomycetes (96), Unclassified Pezizomycetes genus (53)	Uncultured fungus clone (JX323746)	97.27
F18	193	KT991609	Ascomycota (100), Sordariomycetes (100), Microascales (75), Microascaceae (75), *Pseudallescheria* (71)	Uncultured *Pseudallescheria* clone (KM108739)	100
F19	193	KT991610	Ascomycota (100), Sordariomycetes (97), Microascales (81), Microascaceae (80), *Pseudallescheria* (79)	Uncultured *Pseudallescheria* clone (KM108739)	100
F20	212	KT991611	Ascomycota (100), Sordariomycetes (100), Hypocreales (100), Hypocreales_family_incertae_sedis (100), *Stachybotrys* (100)	Uncultured fungus clone (JX348029)	100
F21	202	KT991612	Ascomycota (100), Sordariomycetes (100), Hypocreales (100), Hypocreales_family_incertae_sedis (93), *Fusarium* (93)	*Fusarium equiseti* isolate (KM246255)	100
F22	194	KT991613	Ascomycota (100), Sordariomycetes (99), Hypocreales (99) Hypocreales_family_incertae_sedis (84), *Fusarium* (83)	Uncultured *Fusarium* clone (KP235758)	97.94
F23	193	KT991614	Ascomycota (100), Sordariomycetes (98), Microascales (85), Microascaceae (85), *Pseudallescheria* (82)	Uncultured *Pseudallescheria* clone (KM108739)	100
F24	221	KT991615	Fungi_phylum_incertae_sedis (100), Fungi_class_incertae_sedis (100), Mortierellales (100), Mortierellaceae (99), *Mortierella* (99)	Uncultured fungus clone (GQ866183)	97.26
F25	305	KT991616	Basidiomycota (100), Agaricomycetes (100), Agaricales (100), Psathyrellaceae (100), *Coprinopsis* (100)	*Hormographiella aspergillata* (KP132299)	100
F26	304	KT991617	Basidiomycota (100), Agaricomycetes (100), Agaricales (100), Psathyrellaceae (100), *Coprinopsis* (100)	Uncultured Coprinopsis clone (GQ219811)	100
F27	298	KT991618	Basidiomycota (100), Agaricomycetes (100), Agaricales (100), Psathyrellaceae (99), *Psathyrella* (61)	Uncultured fungus clone (GQ225128)	100
F28	287	KT991619	Basidiomycota (100), Agaricomycetes (100), Agaricales (100), Psathyrellaceae (100), *Coprinopsis* (100)	Uncultured *Coprinopsis* clone (GQ219811)	100
F29	307	KT991620	Basidiomycota (100), Agaricomycetes (100), Agaricales (100), Psathyrellaceae (100), *Coprinopsis* (100)	*Hormographiella aspergillata* (KP132299)	100
F30	204	KT991621	Ascomycota (100) Sordariomycetes (100), Microascales (99), Microascaceae (99), *Pseudallescheria* (99)	Uncultured fungus clone (JX383001)	94.2
F31	204	KT991622	Ascomycota (100), Dothideomycetes (100), Capnodiales (100), Capnodiales_family_incertae_sedis (100), *Davidiella* (53)	*Cladosporium sphaerospermum* (KP174687)	100
F32	245	KT991623	Ascomycota (96), Sordariomycetes (90), Microascales (61), Microascaceae (60), *Scedosporium* (47)	Uncultured fungus clone (JQ989314)	99.59
F33	298	KT991624	Basidiomycota (100), Agaricomycetes (100), Agaricales (100), Psathyrellaceae (99), *Psathyrella* (58)	Uncultured fungus clone (GQ225128)	100
F34	297	KT991625	Basidiomycota (100), Agaricomycetes (100), Agaricales (100), Psathyrellaceae (100), *Coprinopsis* (100)	*Hormographiella aspergillata* (KP132299)	100
F35	308	KT991626	Basidiomycota (100), Agaricomycetes (100), Agaricales (100), Psathyrellaceae (100), *Coprinopsis* (100)	Uncultured *Coprinopsis* clone (GQ219811)	100
F36	305	KT991627	Basidiomycota (100), Agaricomycetes (100), Agaricales (100), Psathyrellaceae (100), *Coprinopsis* (100)	*Hormographiella aspergillata* (KP132299)	100
F37	279	KT991628	Basidiomycota (100), Agaricomycetes (100), Agaricales (100), Psathyrellaceae (100), *Coprinopsis* (100)	*Coprinopsis* sp. (AB499044)	100
F38	206	KT991629	Fungi_phylum_incertae_sedis (99), Fungi_class_incertae_sedis (99), Mortierellales (99), Mortierellaceae (95), *Mortierella* (95)	Uncultured soil fungus clone (JX489813)	100
F39	209	KT991630	Ascomycota (100), Sordariomycetes (100), Hypocreales (100), Hypocreales_family_incertae_sedis (95), *Fusarium* (95)	*Fusarium equiseti* isolate (KM246255)	100
F40	313	KT991631	Basidiomycota (100), Agaricomycetes (100), Agaricales (100), Psathyrellaceae (100), *Coprinellus* (100)	Uncultured fungus clone (JX353314)	100
F41	211	KT991632	Ascomycota (100), Sordariomycetes (100), Hypocreales (100), Hypocreales_family_incertae_sedis (100), *Stachybotrys* (100)	Uncultured *Stachybotrys* clone (KF493978)	100

a *Band numbers correspond to those presented in Figure 3C for fungal samples*.

b *Taxonomic string with bootstrap values (in parentheses), generated in mothur using Findley database*.

c *Closest relative according to INSA (International Nucleotide Sequence Database)*.

d *Percentage sequence similarity with closest INSA using BLAST tool*.

The bacterial and fungal rRNA sequences determined in this study are available at the GenBank under accession numbers KT991569 through KT991632. Each band designation includes a code specifying its origin (ASS, Agricultural Soil Suppressiveness) followed by a number indicating the order in which the sequence was isolated from the gel.

## Discussion

In this study, two suppressive soils to RKN were identified, increasing the list of previous studies reporting this kind of agricultural soil (Stirling et al., 1979; Gaspard et al., 1990; Pyrowolakis et al., 2002; Timper, 2011; Adam et al., 2014). However, as far as the authors know, this is the first report of suppressive soils to RKN in which vegetables are cultivated organically in plastic greenhouses. In addition, despite several studies to identify suppressive soils to RKN, none reported the fluctuation of both nematode densities and fungal egg parasites during the rotation sequences. This is the first comparison of microbial profiles of both suppressive and non-suppressive soils to be published.

The antagonistic potential of agricultural soils, defined as its capacity to prevent or reduce the spread of pathogens by biotic factors, is a product of the capacity of the microbial antagonists to survive the agronomic practices and their ability to limit the damage caused by the pathogens (Sikora, 1992). It is widely accepted that high levels of suppressiveness to plant parasitic nematodes are only achieved under perennial crops or monoculture in which soil perturbation practices are low (Baker and Cook, 1974). However, this study shows that high levels of soil suppressiveness can be also achieved in highly perturbed crop systems, probably due to the confluence of favorable interactions between plant-RKN-antagonists, cultural practices and abiotic factors. Both sites, M10.23 and M10.55, were located in the same cropping area, with similar agro-climatic conditions, but differing in crop management. In both sites, RKN were detected in soil and in roots at the beginning of the study. However, nematode densities decreased to near and below detectable levels in soil and roots, respectively, at the end of the study in site M10.23, but not in site M10.55, in which RKN was always detected. Agricultural practices such as crop rotation, tillage and organic amendments have been proved to influence the antagonistic potential of soil (Sikora, 1992; Kerry and Bourne, 1996; Westphal and Becker, 2001; Janvier et al., 2007; Timper, 2011), and could be the reason for the results of this study. For instance, site M10.23 was fertilized with a mixture of sheep and chicken manure but only sheep manure was used at M10.55. Chicken manure has been reported to suppress RKN infection and reproduction on several crops (Kaplan and Noe, 1993; Riegel and Noe, 2007), but there is still limited information about the suppressive capacity of sheep manure.

In site M10.23, the nematode was able to reproduce on susceptible crops cultivated during spring-summer, in which fungal RKN egg parasites were isolated, mainly *P. chlamydosporia*. The highest percentage of fungal egg parasitism was recorded on zucchini, which ranged from 30 to 78% in the four plots (mean of 54.7%). At the end of this crop, galling index ranged from 0 to 4, less than expected considering a *Pi* of 2951 J2 250 cm^−3^ of soil, and in which the nematode completed three generations according to thermal requirements of *M. incognita* (Vela et al., 2014). Vela et al. (2014) recorded galling indexes of 2.6 and 5.1 on zucchini cultivated in plastic greenhouse, with *Pi* of 222 and 594 J2 250 cm^−3^ of soil, respectively, and in which nematodes completed two generations. *P. chlamydosporia* is a fungal egg parasite that affects the increase of nematode inoculum (J2) and consequently reduces disease severity when more than one generation occurs, because emerged J2 from non-parasitized eggs are able to invade roots (Bailey et al., 2008). Results from the pot test conducted for just one nematode generation showed differences in disease severity between sterilized and non-sterilized soils, indicating that other microorganisms could be involved in soil suppressiveness. Fungal and bacterial DNA sequenced from soil DGGE revealed the presence of several species that can affect nematodes by the production of active toxins against RKN J2 such *Stachybotrys* spp. *Cladosporium* spp. (Qureshi et al., 2012), and *Flavobacterium* spp. (McClure, 1989); by inducing the activity of other nematode antagonists, such *Chryseobacterium* spp. that induce trap formation in *Arthobotrys oligospora* (Li et al., 2011); by suppressing disease severity, such *Chryseobacterium* spp. (Liu et al., 2014); or by parasitizing RKN eggs, such *Fusarium equiseti* and *Cladosporium* spp. (Giné et al., 2012). The growing media used in this study did not allow bacterial isolation. Thus, the use of culture independent methods is necessary to complement the information obtained by traditional culture dependent ones in order to know the composition and function of microbial communities and their putative contribution to soil suppressiveness.

At the end of autumn-winter crops, no galls were observed and no eggs were extracted from roots. Soil temperatures influence the movement of J2 in soil, root penetration and infection, development and reproduction of RKN. Minimum and maximum soil temperatures at planting autumn-winter crops were 8.3 and 17.3°C (mean 12.8°C), temperatures below the minimum activity threshold of J2 (Roberts et al., 1981). Thus, roots could escape infection.

In site M10.55, *Meloidogyne* was detected in soil and roots of each crop. Nematode densities fluctuated during the cropping season as well as *P. chlamydosporia*, the only fungal species isolated from eggs. Highest nematode densities and levels of egg parasitism were recorded at the end of cucumber and zucchini cultivated in spring-summer and summer-winter, respectively. *Meloidogyne* completed two generations on cucumber and three on zucchini according to RKN thermal requirements on these crops (Giné et al., 2014; Vela et al., 2014). However, disease severity was less than expected, as occurred in M10.23. Soil microbial profiles showed the occurrence of Cyanobacteria, able to suppress RKN densities and disease severity (Khan et al., 2007), and the fungi, *F. equiseti*, and *Preussia* spp, which have been reported as egg parasite of *Heterodera schachtii* (Saleh and Qadri, 1990). Results of pot experiments suggest that the only active antagonist of RKN was *P. chlamydosporia* because despite high percentage of egg parasitism was recorded, there was no reduction on disease severity after completion of one nematode generation, but it did in field conditions in which the nematode completed more than one.

Despite resistant tomato cultivars suppressed nematode densities and disease severity, as previously reported in plastic greenhouses in Spain (Sorribas et al., 2005; Talavera et al., 2009), *P. chlamydosporia* was also isolated, but the percentage of egg parasitism decreased compared to those on susceptible crops. A positive relation (*r* = 0.89, *P* = 0.042) between egg density on roots (logarithm) and percentage of egg parasitism was found demonstrating the density dependent relationship, as previously stated (Bourne and Kerry, 1998).

Lettuce cultivated from November to March or September to November reduced or maintained nematode densities in soil, but the number of eggs on roots was fewer when planted in November than in September. Absolute minimum and maximum soil temperatures from November to March were 5.1°C and 21.0°C, and 16.5°C and 29.1°C from September to November. Thus, in lettuce planted in September, RKN was able to accumulate enough degree days to complete its life cycle, to produce more eggs and to maintain densities in soil than when cultivated from November to March in which no eggs were produced and fewer nematodes were recovered from soil. The date of planting has an important repercussion in the life cycle of *Meloidogyne* because after root penetration, the nematode needs to accumulate a minimum number of degree days over a specific temperature threshold to complete its life cycle, otherwise, the crop will act as a trap crop. Some crops as lettuce, radish, and arugula have been used as trap crops (Cuadra et al., 2000; Melakeberhan et al., 2006). In north-eastern Spain, lettuce acted as a trap crop when it was transplanted in middle October or November, but not in September when the nematode was able to accumulate enough degree days to produce eggs (Ornat and Sorribas, 2008).

The cover crop of mustard cv. Caliente 119 (a blend of white mustard, *Sinapis alba*, and Indian mustard, *Brassica juncea*) was used as green manure. After its incorporation into the soil, nematode densities dropped considerably as well as percentage of fungal egg parasitism at the end of the following lettuce crop. Mustard cv. Caliente 119 has been shown effective against plant-parasitic nematodes and soil-borne fungi (Potter et al., 1998; Charron and Sams, 1999; Friberg et al., 2009). Nevertheless, *P. chlamydosporia* survived, being recovered after the following resistant tomato crop in 2013.

DGGE fingerprints revealed the occurrence of fungal and bacterial species that have been reported associated with the cuticle of RKN J2 or egg masses, or *Heterodera glycines* cysts (Nour et al., 2003; Adam et al., 2014; Cao et al., 2015), but the effect of the majority of them on viability of the nematode is unknown. Some of them such as *Mortierella* spp., Sphingobacteriales, and *Methylobacterium* spp. (reported associated with the J2 cuticle), and *Flexibacter* spp. (associated with the cysts of *H. glycines*) were identified in non-suppressive and one or both suppressive soils. *Davidiella* spp. reported associated with the J2 cuticle was only identified in non-suppressive soil M10.33; *Sphingobacterium* spp., reported associated with *H. glycines* cysts was only identified in M10.55. *Steroidobacter* spp. and *Lysobacter* spp. were reported associated with RKN egg masses and were only identified in M10.23. Several species of *Lysobacter* spp., affect egg hatching of *Meloidogyne* sp. (Chen et al., 2006; Lee et al., 2014) and reduced disease severity in pot tests (Lee et al., 2013).

Diversity indices were similar for both suppressive and non-suppressive soils. In fact, suppressiveness is more related to microbial functionality than diversity. In both suppressive soils, *P. chlamydosporia* was the only and most prevalent fungal egg parasites recovered from RKN eggs throughout the study and deemed to be one of the factors responsible for soil suppressiveness in M10.23, and the most responsible in M10.55. In this study, a density dependent relationship between percent of egg parasitism and density of eggs in roots was found, according to that reported by Bourne and Kerry (1998). Moreover, great variability in virulence of several isolates coming from the same soil was also found. It is known that isolates of *P. chlamydosporia* from the same or different soils differ greatly in growth, development and virulence, in their saprophytic and parasitic ability, and in their ability to colonize the plant rhizosphere (Kerry and Hirsch, 2011). Thus, the environmental plasticity and variability in the virulence showed in this study could be a strategy to persist in a given site, even at low densities. *P. chlamydosporia* was fully adapted to these soil environments and agronomical management practices. It was recovered from eggs in field and pot experiments in site M10.55, or in pot experiments from non-sterilized soils despite no eggs being produced in the majority of crops in field conditions in site M10.23. This plasticity could explain why *P. chlamydosporia* has been found more frequently in the last years in north-eastern Spain, since integrated and organic production systems have been increasingly implemented by growers (Verdejo-Lucas et al., 2002; Giné et al., 2014).

This research provides new information about the antagonistic potential of soils against RKN in two sites used for commercial production of vegetables under organic standards in plastic greenhouse during two growing seasons. *P. chlamydosporia* was the main biotic factor responsible of suppressiveness in site M10.55, because it was the only fungal species recovered from RKN eggs in the field study and pot experiments, and no other antagonist species or effects on RKN were identified by DNA sequencing from DGGE or in pot experiments. However, in M10.23, RKN suppressiveness could be attributed to a combination of microbes, because despite *P. chlamydosporia* was isolated from eggs, some other microorganism with antagonistic effect against the nematode were identified by DGGE and results from pot test agree with their mode of action. Besides the biotic factors identified in both sites, a combination of several agronomic practices such as crop rotation, including RKN resistant cultivars and cover crop as green manure, the addition of organic amendments, and date of planting, can contribute to prevent nematode build-up. These findings will lead to further studies deep in the knowledge of the relations between microbial communities and crop management that achieve soil suppressiveness, in order to design strategies to improve the antagonistic potential of soil.

## Author contributions

This work is a part of the Ph.D. thesis of AG, directed by FS, AG, and FS were involved in field studies, pot tests, isolation and identification of fungal egg parasites, and pathogenicity tests. MC, MM, and NG we involved in DGGE analysis. All authors contributed to the writing of the manuscript and approved submission.

### Conflict of interest statement

The authors declare that the research was conducted in the absence of any commercial or financial relationships that could be construed as a potential conflict of interest.

## References

[B1] Adam M. Westphal A. Hallmann J. Heuer H. (2014). Specific microbial attachment to root knot nematodes in suppressive soil. Appl. Environ. Microbiol. 80, 2679–2686. 10.1128/AEM.03905-13 24532076 PMC3993313

[B2] Bailey D. J. Biran G. L. Kerry B. R. Gilligan C. A. (2008). Pathozone dynamics of *Meloidogyne incognita* in the rhizosphere of tomato plants in the presence and absence of the nematophagous fungus, *Pochonia chlamydosporia*. Plant Pathol. 57, 354–362. 10.1111/j.1365-3059.2007.01776.x

[B3] Baker K. Cook R. J. (1974). Biological Control of Plant Pathogens. San Francisco, CA: WH Freeman and Company.

[B4] Bourne J. M. Kerry B. R. (1998). Effect of the host plant on the efficacy of *Verticillium chlamydosporium* as a biological control agent of root-knot nematodes at different nematode densities and fungal application rates. Soil Biol. Biochem. 31, 75–84. 10.1016/S0038-0717(98)00107-2

[B5] Cao Y. Tian B. Ji X. Shang S. Lu C. Zhang K. (2015). Associated bacteria of different life stages of *Meloidogyne incognita* using pyrosequencing-based analysis. J. Basic Microbiol. 55, 950–960. 10.1002/jobm.201400816 25809195

[B6] Charron C. Sams C. (1999). Inhibition of *Pythium ultimum* and *Rhizoctonia solani* by shredded leaves of *Brassica* species. J. Am. Soc. Hortic. Sci. 124, 462–467.

[B7] Chen J. Moore W. H. Yuen G. Y. Kobayashi D. Caswell-Chen E. P. (2006). Influence of *Lysobacter enzymogenes* strain C3 on nematodes. J. Nematol. 38, 233–239. 19259452 PMC2586455

[B8] Chen S. (2007). Suppression of *Heterodera glycines* in soils from fields with long-term soybean monoculture. Biocontrol Sci. Technol. 17, 125–134. 10.1080/09583150600937121

[B9] Cuadra R. Cruz X. Fajardo J. L. (2000). Cultivos de ciclo corto como plantas trampas para el control del nematodo agallador. Nematropica 30, 241–246.

[B10] de Leij F. A. A. M. Kerry B. R. (1991). The nematophagous fungus, *Verticillium chlamydosporium*, as a potential biological control agent for *Meloidogyne arenaria*. Rev. Nematol. 14, 157–164.

[B11] Edgar R. C. Haas B. J. Clemente J. C. Quince C. Knight R. (2011). UCHIME improves sensitivity and speed of chimera detection. Bioinformatics 27, 2194–2200. 10.1093/bioinformatics/btr381 21700674 PMC3150044

[B12] Ferris H. Roberts P. A. Thomason I. J. (1985). Nematodes, in Integrated Pest Management for Tomatoes (University of California; Statewide Integrated Pest Management Project; Division of Agriculture and Natural Resources), 60–65.

[B13] Friberg H. Edel-Hermann V. Faivre C. Gautheron N. Fayolle L. Faloya V. (2009). Cause and duration of mustard incorporation effects on soil-borne plant pathogenic fungi. Soil Biol. Biochem. 41, 2075–2084. 10.1016/j.soilbio.2009.07.017

[B14] Gams W. (1988). A contribution to the knowledge of nematophagous species of Verticillium. Neth. J. Plant Pathol. 94, 123–148. 10.1007/BF01978003

[B15] Garbeva P. van Veen J. A. Van Elsas J. D. (2004). Microbial diversity in soil: selection of microbial populations by plant and soil type and implications for disease suppressiveness. Annu. Rev. Phytopathol. 42, 243–270. 10.1146/annurev.phyto.42.012604.135455 15283667

[B16] Gardes M. Bruns T. D. (1993). ITS primers with enhanced specificity for basidiomycetes - application to the identification of mycorrhizae and rusts. Mol. Ecol. 2, 113–118. 10.1111/j.1365-294X.1993.tb00005.x 8180733

[B17] Gaspard J. T. Jaffee B. A. Ferris H. (1990). M*eloidogyne incognita* survival in soil infested with *Paecilomyces lilacinus* and *Verticillium chlamydosporium*. J. Nematol. 22, 176–181. 19287707 PMC2619027

[B18] Giné A. Bonmatí M. Sarro A. Stchiegel A. Valero J. Ornat C. (2012). Natural occurrence of fungal egg parasites of root-knot nematodes, *Meloidogyne* spp. in organic and integrated vegetable production systems in Spain. Biocontrol 58, 407–416. 10.1007/s10526-012-9495-6

[B19] Giné A. López-Gómez M. Vela M. D. Ornat C. Talavera M. Verdejo-Lucas S. (2014). Thermal requirements and population dynamics of root-knot nematodes on cucumber and yield losses under protected cultivation. Plant Pathol. 63, 1446–1453. 10.1111/ppa.12217

[B20] Hussey R. Barker K. (1973). Comparison of methods of collecting inocula of *Meloidogyne* spp., including a new technique. Plant Dis. Report 57, 1025–1028.

[B21] Janvier C. Villeneuve F. Alabouvette C. Edel-Hermann V. Mateille T. Steinberg C. (2007). Soil health through soil disease suppression: which strategy from descriptors to indicators? Soil Biol. Biochem. 39, 1–23. 10.1016/j.soilbio.2006.07.001

[B22] Jenkins W. (1964). A rapid centrifugal-flotation technique for separating nematodes from soil. Plant Dis. Report 48:692.

[B23] Kaplan M. Noe J. P. (1993). Effects of chicken-excrement amendments on *Meloidogyne arenaria*. J. Nematol. 25, 71–77. 19279745 PMC2619344

[B24] Kerry B. (1980). Biocontrol: fungal parasites of female cyst nematodes. J. Nematol. 12, 253–259. 19300700 PMC2618033

[B25] Kerry B. Bourne J. (1996). The importance of rhizosphere interactions in the biological control of plant parasitic nematodes—a case study using *Verticillium chlamydosporium*. Pestic. Sci. 47, 69–75.

[B26] Kerry B. Hirsch P. (2011). Ecology of *Pochonia chlamydosporia* in the rhizosphere at the population, whole organism and molecular scales, in Biogical Control of Plant-Parasitic Nematodes, eds Davies K. Spiegel Y. (Dordrecht: Springer), 171–182.

[B27] Khan Z. Kim Y. H. Kim S. G. Kim H. W. (2007). Observations on the suppression of root-knot nematode (*Meloidogyne arenaria*) on tomato byincorporation of cyanobacterial powder (*Oscillatoria chlorina*) into potting field soil. Bioresour. Technol. 98, 69–73. 10.1016/j.biortech.2005.11.029 16458501

[B28] Lee Y. S. Anees M. Hyun H. N. Kim K. Y. (2013). Biocontrol potential of *Lysobacter antibioticus* HS124 against the root-knot nematode, *Meloidogyne incognita*, causing disease in *tomato*. Nematology 15, 545–555. 10.1163/15685411-00002700

[B29] Lee Y. S. Nguyen X. H. Moon J. H. (2014). Ovicidal activity of lactic acid produced by *Lysobacter capsici* YS1215 on eggs of root-knot nematode, *Meloidogyne incognita*. J. Microbiol. Biotech. 24, 1510–1515. 10.4014/jmb.1405.05014 25085571

[B30] Li L. Ma M. Liu Y. Zhou J. Qu Q. Lu K.. (2011). Induction of trap formation in nematode-trapping fungi by a bacterium. FEMS Microbiol. Lett. 322, 157–165. 10.1111/j.1574-6968.2011.02351.x 21722172

[B31] Liu H.-X. Li S.-M. Luo Y.-M. Luo L.-X. Li J.-Q. Guo J.-H. (2014). Biological control of Ralstonia wilt, Phytophthora blight, Meloidogyne root-knot on bell pepper by the combination of *Bacillus subtilis* AR12, *Bacillus subtilis* SM21 and *Chryseobacterium* sp. R89. Eur. J. Plant Pathol. 139, 107–116. 10.1007/s10658-013-0369-2

[B32] López-Gómez M. Gine A. Vela M. D. Ornat C. Sorribas F. J. Talavera M. (2014). Damage functions and thermal requirements of *Meloidogyne javanica* and *Meloidogyne incognita* on watermelon. Ann. Appl. Biol. 165, 466–473. 10.1111/aab.12154

[B33] Lopez-Llorca L. Duncan J. (1986). New media for the estimation of fungal infection in eggs of the cereal cyst nematode, *Heterodera avenae* Woll. Nematologica 32, 486–489. 10.1163/187529286X00354

[B34] Lopez-Llorca L. Bordallo J. Salinas J. Monfort E. López-Serna M. (2002). Use of light and scanning electron microscopy to examine colonisation of barley rhizosphere by the nematophagous fungus *Verticillium chlamydosporium*. Micron 33, 61–67. 10.1016/S0968-4328(00)00070-6 11473815

[B35] Maidak B. L. Cole J. R. Lilburn T. G. Parker C. T. Jr. Saxman P. R. Farris R. J.. (2001). The RDP-II (Ribosomal Database Project). Nucleic Acids Res. 29, 173–174. 10.1093/nar/29.1.173 11125082 PMC29785

[B36] McClure M. A. (1989). Neoplastic growths in preparasitic juveniles of *Meloidogyne incognita*. J. Nematol. 21, 427–430. 19287632 PMC2618938

[B37] Melakeberhan H. Xu A. Kravchenko A. Mennan S. Riga E. (2006). Potential use of arugula (*Eruca sativa* L.) as a trap crop for *Meloidogyne hapla*. Nematology 8, 793–799. 10.1163/156854106778877884

[B38] Melgarejo P. García-Jiménez J. Jordá M. C. López M. M. Andrés M. F. Duran-Vila N. (2010). Patógenos de plantas descritos en España, 2nd Edn. Madrid: Ministerio de Medio Ambiente y Medio Rural y Marino.

[B39] Muyzer G. Brinkhoff T. Nübel U. Santegoeds C. Schäfer H. Wawer C. (2004). Denaturing gradient gel electrophoresis (DGGE) in microbial ecology, in Molecular Microbial Ecology Manual, Vol. 1–2, 2nd Edn., eds Kowalchuk G. A. de Bruijn F. J. Head I. M. Akkermans A. D. van Elsas J. D. (Dordrecht: Springer), 743–769.

[B40] Nour S. M. Lawrence J. R. Zhu H. Swerhone G. D. W. Welsh M. Welacky T. W.. (2003). Bacteria associated with cysts of the soybean cyst nematode (*Heterodera glycines*). Appl. Environ. Microbiol. 69, 607–615. 10.1128/AEM.69.1.607-615.2003 12514048 PMC152414

[B41] Olivares-Bernabeu C. M. López-Llorca L. V. (2002). Fungal egg-parasites of plant-parasitic nematodes from Spanish soils. Rev. Iberoam. Micol. 19, 104–110. 12828513

[B42] Ornat C. Sorribas F. (2008). Integrated management of root-knot nematodes in mediterranean horticultural crops, in Integrated Management and Biocontrol of Vegetable and Grain Crops Nematodes, eds Ciancio A. Mukerji K. G. (Dordrecht: Springer), 295–319.

[B43] Potter M. J. Davies K. Rathjen A. J. (1998). Suppressive impact of glucosinolates in Brassica vegetative tissues on root lesion nematode *Pratylenchus neglectus*. J. Chem. Ecol. 24, 67–80. 10.1023/A:1022336812240

[B44] Pyrowolakis A. Westphal A. Sikora R. A. Ole Becker J. (2002). Identification of root-knot nematode suppressive soils. Appl. Soil Ecol. 19, 51–56. 10.1016/S0929-1393(01)00170-6

[B45] Qureshi S. Ruqqia A. Sultana V. Ara J. Ehteshamul-Haque S. (2012). Nematicidal potential of culture filtrates of soil fungi associated with rhizosphere and rhizoplane of cultivated and wild plants. Pak. J. Bot. 44, 1041–1046.

[B46] Riegel C. Noe J. P. (2007). Chicken litter soil amendment effects on soilborne microbes and *Meloidogyne incognita* on cotton. Plant Dis. 84, 1275–1281. 10.1094/PDIS.2000.84.12.1275 30831867

[B47] Roberts P. A. Van Gundy S. D. McKinney H. E. (1981). Effects of soil temperature and planting date of wheat on *Meloidogyne incognita* reproduction, soil populations, and grain yield. J. Nematol. 13, 338–345. 19300773 PMC2618102

[B48] Saleh H. M. Qadri A. N. (1990). Fungi associated with *Heterodera schachtii* (Nematoda) in Jordan Ii) Effect on *H. schachtii* and *Meloidogyne javanica*. Nematologica 36, 104–113. 10.1163/002925990X00077

[B49] Sasser J. Freckman D. (1987). A world perspective on nematology: the role of the society in Vistas on Nematology, eds Veech J. A. Dickson D. W. (Hyattsville, MD: Society of Nematologists), 7–14.

[B50] Schäfer H. Muyzer G. (2001). Denaturing gradient gel electrophoresis in marine microbial ecology. Mar. Microbiol. 30, 425 10.1016/S0580-9517(01)30057-0

[B51] Sikora R. A. (1992). Management of the antagonistic potential in agricultural ecosystems for the biological control of plant parasitic nematodes. Annu. Rev. Phytopathol. 30, 245–270. 10.1146/annurev.py.30.090192.001333

[B52] Smalla K. Heuer H. (2006). How to assess the abundance and diversity of mobile genetic elements in soil bacterial communities? in Nucleic Acids and Proteins in Soil, eds Nannipieri P. Smalla K. (Berlin; Heidelberg: Springer), 313–330.

[B53] Sorribas F. J. Ornat C. Galeano M. Verdejo-Lucas S. (2003). Evaluation of a native and introduced isolate of *Pochonia chlamydosporia* against *Meloidogyne javanica*. Biocontrol Sci. Technol. 13, 707–714. 10.1080/09583150310001606282

[B54] Sorribas F. J. Ornat C. Verdejo-Lucas S. Galeano M. Valero J. (2005). Effectiveness and profitability of the Mi-resistant tomatoes to control root-knot nematodes. Eur. J. Plant Pathol. 111, 29–38. 10.1007/s10658-004-1982-x

[B55] Stirling G. R. McKenry M. V. Mankau R. (1979). Biological control [mainly by the fungus *Dactylella oviparasitica*] of root-knot nematodes (*Meloidogyne* spp.) on peach. Phytopathology 69, 806–809. 10.1094/Phyto-69-806

[B56] Talavera M. Sayadi S. Verdejo-Lucas S. (2012). Perception of the impact of root-knot nematode-induced diseases in horticultural protected crops of south-eastern Spain. Nematology 14, 517–527. 10.1163/156854112X635850

[B57] Talavera M. Verdejo-Lucas S. Ornat C. Torres J. Vela M. D. Macias F. J. (2009). Crop rotations with Mi gene resistant and susceptible tomato cultivars for management of root-knot nematodes in plastic houses. Crop Prot. 28, 662–667. 10.1016/j.cropro.2009.03.015

[B58] Tatusova T. A. Madden T. L. (1999). BLAST 2 Sequences, a new tool for comparing protein and nucleotide sequences. FEMS Microbiol. Lett. 174, 247–250. 10.1111/j.1574-6968.1999.tb13575.x 10339815

[B59] Timper P. (2011). Utilization of biological control for managing plant-parasitic nematodes, in Biolologia Control Plant-Parasitic Nematodes, eds Davies K. Spiegel Y. (Dordrecht: Springer), 259–289.

[B60] Vela M. D. Giné A. López-Gómez M. Sorribas F. J. Ornat C. Verdejo-Lucas S. (2014). Thermal time requirements of root-knot nematodes on zucchini-squash and population dynamics with associated yield losses on spring and autumn cropping cycles. Eur. J. Plant Pathol. 140, 481–490. 10.1007/s10658-014-0482-x

[B61] Verdejo S. Jaffee B. A. Mankau R. (1988). Reproduction of *Meloidogyne javanica* on plant roots genetically transformed by *Agrobacterium rhizogenes*. J. Nematol. 20, 599–604. 19290260 PMC2618848

[B62] Verdejo-Lucas S. Blanco M. Talavera M. Stchigel A. M. Sorribas F. J. (2013). Fungi recovered from root-knot nematodes infecting vegetables under protected cultivation. Biocontrol Sci. Techn. 23, 277–287. 10.1080/09583157.2012.756459

[B63] Verdejo-Lucas S. Espanol M. Ornat C. Sorribas F. J. (1997). Occurrence of *Pasteuria* spp. in northeastern Spain. Nematol. Mediterranea 25, 109–112.

[B64] Verdejo-Lucas S. Ornat C. Sorribas F. J. Stchiegel A. (2002). Species of root-knot nematodes and fungal egg parasites recovered from vegetables in Almería and Barcelona, Spain. J. Nematol. 34, 405–408. 19265964 PMC2620581

[B65] Verdejo-Lucas S. Sorribas J. Puigdomènech P. (1994). Pérdidas de producción en lechuga y tomate causadas por *Meloidogyne javanica* en invernadero. Investig. Agrar. 2, 395–400.

[B66] Weller D. M. Raaijmakers J. M. Gardener B. B. M. Thomashow L. S. (2002). Microbial populations responsible for specific soil suppressiveness to plant pathogens. Annu. Rev. Phytopathol. 40, 309–348. 10.1146/annurev.phyto.40.030402.110010 12147763

[B67] Westphal A. Becker J. (2001). Components of soil suppressiveness against *Heterodera schachtii*. Soil Biol. Biochem. 33, 9–16. 10.1016/S0038-0717(00)00108-5

[B68] Westphal A. Becker J. O. (1999). Biological suppression and natural population decline of *Heterodera schachtii* in a California field. Phytopathology 89, 434–440. 18944757 10.1094/PHYTO.1999.89.5.434

[B69] White T. Bruns T. Lee S. Taylor J. (1990). Amplification and direct sequencing of fungal ribosomal RNA genes for phylogenetics. PCR Protoc. 18, 315–322. 10.1016/b978-0-12-372180-8.50042-1

[B70] Whitehead A. G. Hemming J. R. (1965). A comparison of some quantitative methods of extracting small vermiform nematodes from soil. Ann. Appl. Biol. 55, 25–38. 10.1111/j.1744-7348.1965.tb07864.x

[B71] Zeck W. (1971). Rating scheme for field evaluation of root-knot nematode infestations. Pflanzenschutz Nachrichten 24, 141–144.

